# S1P promotes IL-6 expression in osteoblasts through the PI3K, MEK/ERK and NF-κB signaling pathways

**DOI:** 10.7150/ijms.44612

**Published:** 2020-05-18

**Authors:** Sung-Lin Hu, Chien-Chung Huang, Tzu-Ting Tzeng, Shan-Chi Liu, Chun-Hao Tsai, Yi-Chin Fong, Chih-Hsin Tang

**Affiliations:** 1 School of Medicine, China Medical University, Taichung, Taiwan; 2 Department of Family Medicine, China Medical University Hsinchu Hospital, Hsinchu, Taiwan; 3 Division of Immunology and Rheumatology, Department of Internal Medicine, China Medical University Hospital, Taichung, Taiwan; 4 Graduate Institute of Biomedical Sciences, China Medical University, Taichung, Taiwan; 5 Department of Medical Education and Research, China Medical University Beigang Hospital, Yunlin, Taiwan; 6 Department of Sports Medicine, College of Health Care, China Medical University, Taichung, Taiwan; 7 Department of Orthopedic Surgery, China Medical University Hospital, Taichung, Taiwan; 8 Department of Orthopedic Surgery, China Medical University Beigang Hospital, Yunlin, Taiwan; 9 Chinese Medicine Research Center, China Medical University, Taichung, Taiwan; 10 Department of Biotechnology, College of Health Science, Asia University, Taichung, Taiwan

**Keywords:** S1P, IL-6, Osteoblasts, Arthritis

## Abstract

Rheumatoid arthritis (RA) is a systemic autoimmune inflammatory disease, in which the immune system attacks joint tissue. Interleukin (IL)-6 is a key proinflammatory cytokine in RA progression. Sphingosine-1-phosphate (S1P), a platelet-derived lysophospholipid mediator, reportedly regulates osteoimmunology. Here, we examined the effects of S1P on IL-6 expression in osteoblasts. Our results and records from the Gene Expression Omnibus (GEO) database demonstrate higher levels of IL-6 in patients with RA compared with those with osteoarthritis. Stimulation of osteoblasts with S1P increased mRNA and protein expression of IL-6. PI3K, MEK, ERK and NF-κB inhibitors and their small interfering RNAs (siRNAs) reduced S1P-promoted IL-6 expression. S1P also facilitated PI3K, MEK/ERK and NF-κB signaling cascades. Our results indicate that S1P promotes the expression of IL-6 in osteoblasts via the PI3K, MEK/ERK and NF-κB signaling pathways.

## Introduction

Rheumatoid arthritis (RA) is a systemic autoimmune inflammatory disease, in which the immune system attacks joint tissue 1, 2. An inflammatory response, pannus development, synovial swelling, joint stiffness and articular cartilage degradation are major symptoms of RA 3. Thus, much RA research has concentrated on synovial inflammation and cartilage destruction 4. Few reports have focused on the effects of subchondral bone erosions in RA pathogenesis 5-7. Accumulating evidence suggests that osteoblasts, the most abundant cell type in subchondral bone, play an important role in cartilage pathology during the development of RA 8, 9. Thus, elucidation of the molecular mechanism of osteoblasts is a critical issue for RA treatment.

Interleukin (IL)-6, a T-cell-derived cytokine that promotes the maturation of B cells into antibody-secreting cells, involves multiple biological activities and plays a critical role in innate and acquired immune responses, cancer progression and metastasis, inflammatory reactions, as well as osteoclast generation 10, and is well recognized as an important proinflammatory cytokine during RA progression 11, 12. The therapeutic success of the IL-6 inhibitor tocilizumab in RA has encouraged the development of other IL-6 inhibitors, only one of which (sarilumab) has so far received EU and US regulatory approval for the treatment of RA 13.

Sphingosine-1-phosphate (S1P), a platelet-derived lysophospholipid mediator, reduces platelet-derived growth factor (PDGF)-promoted chemotaxis and cellular Rac activation 14. The S1P/S1P receptor axis regulates several biologic functions such as tumor invasion and progression, angiogenesis and vasculogenesis as well as skeletal muscle and nervous system degeneration 15-17. Interestingly, deleting the S1P_2_ receptor promotes murine embryonic fibroblast migration towards S1P and also PDGF, which stimulates S1P production; S1P_2_ deletion also increases the enzymatic expression and activity of sphingosine kinase 1 (SphK1), which is responsible for producing S1P 18. In addition, S1P/S1P_1_ signaling reportedly regulates osteoimmunology 19 and upregulation of the S1P receptor in synovial tissues has found in the collagen-induced arthritis model 20. However, the role of S1P in IL-6 expression and RA pathogenesis is uncertain. We describe how we found that S1P promotes IL-6 expression in human osteoblasts and also involvement of the PI3K, MEK/ERK and NF-κB signaling pathways in S1P-promoted IL-6 production.

## Materials and Methods

### Materials

We obtained antibodies against p-PI3K, p-MEK, p-ERK, p-p65, PI3K, MEK, ERK, p65 and β-actin from Santa Cruz Biotechnology (CA, USA). S1P was purchased from Avanti Polar Lipid Inc. (Alabaster, AL, USA). ON-TARGETplus siRNAs were purchased from Dharmacon Research (Lafayette, CO, USA). Gibco-BRL Life Technologies (Grand Island, NY, USA) supplied fetal bovine serum (FBS) and all other cell culture reagents. Promega (Madison, WI, USA) supplied the pSV-β-galactosidase vector and luciferase assay kits. The κB luciferase plasmid was purchased from Stratagene (La Jolla, CA). The human IL-6 promoter constructs were provided by Dr. Oliver Eickelberg (Department of Medicine II, University of Giessen, Giessen, Germany). All other chemicals or inhibitors were purchased from Sigma-Aldrich (St. Louis, MO, USA).

### Human synovial fluids

We obtained approval for this study from the local ethics committee (the Institutional Review Board of China Medical University Hospital) and all patients gave written informed consent before participating in this study. Abnormal synovial fluids were obtained from patients undergoing total knee arthroplasty for osteoarthritis (OA) or RA.

### Analysis of the Gene Expression Omnibus (GEO) database

Gene expression profile records were obtained from the GEO database for analysis of IL-6 expression in OA and RA tissue samples 21.

### Cell culture

Osteoblast-like cell lines MG-63 and MC3T3-E1 were purchased from American Type Culture Collection (Manassas, VA, USA). Cells were cultured in MEM supplemented with 10% FBS and antibiotics then maintained in a humidified incubator at 37°C in 5% CO_2_.

### Western blot analysis

Extracted proteins were resolved by SDS-PAGE and transferred to Immobilon^®^ PVDF membranes. Western blot analysis was performed according to our previous reports 22-24.

### Quantitative real-time PCR (qPCR)

Total RNA was extracted from osteoblasts using TRIzol reagent. qPCR analysis was conducted according to an established protocol 25-27.

### Enzyme-Linked Immunosorbent (ELISA) assay

Osteoblasts were treated with S1P alone for 24 h or pretreated with pharmacological inhibitors, followed by 24 h of S1P stimulation. The conditioned medium (CM) was collected and quantified for IL-6 levels using an IL-6 ELISA kit (Peprotech, Rocky Hill, NJ, USA), as per the manufacturer's protocol.

### Luciferase reporter assay

Osteoblasts were co-transfected with 0.8 μg IL-6 or κB-luciferase reporter gene construct and 0.4 μg β-galactosidase using Lipofectamine 2000, as per the manufacturer's instructions. After 24 h of transfection, the cells were exposed to S1P. Luciferase activity was determined using the luciferase assay kit 28-30.

### Statistics

All values are presented as the mean ± standard deviation (SD). Differences between the two experimental groups were assessed for significance using the Student's *t*-test. The difference was considered to be significant if the *p* value was < 0.05.

## Results

### Upregulation of IL-6 expression in RA synovial fluid

**Figure 1:**
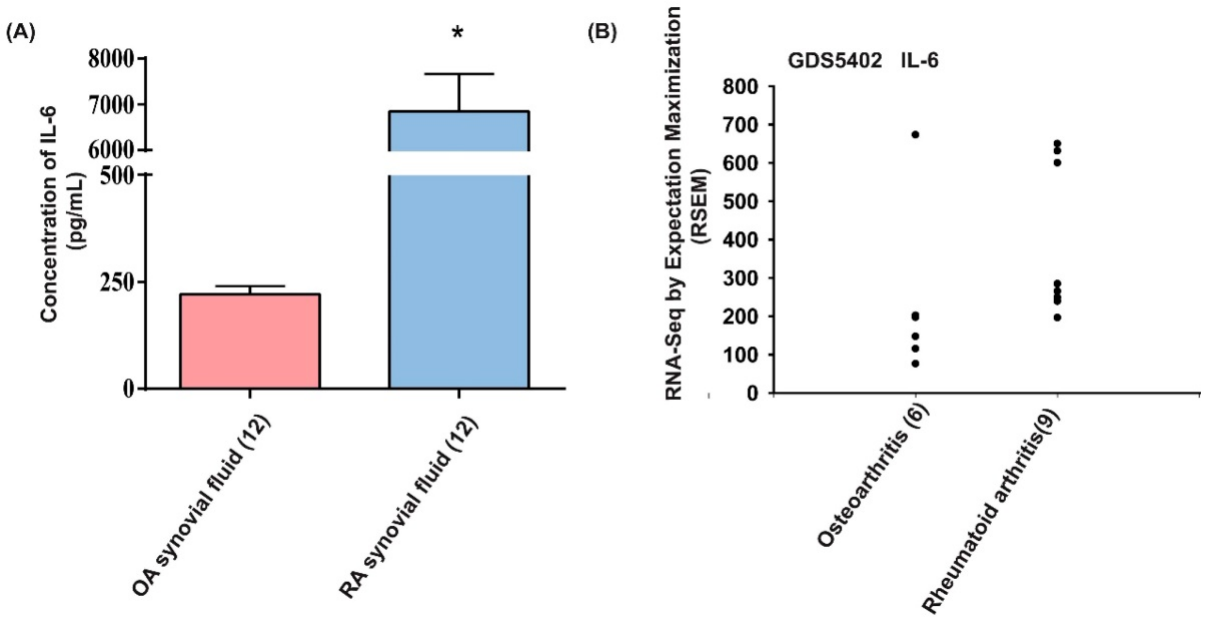
** IL-6 levels are upregulated in RA patients.** (A) IL-6 levels in synovial fluid from patients with OA and RA were quantified using the ELISA assay. (B) Levels of IL-6 expression in human RA and OA tissue samples obtained from the GEO database. Data represent the mean ± S.D.

The proinflammatory role of IL-6 is well understood during RA pathogenesis 13. To further confirm the role of IL-6 in RA angiogenesis, we first analyzed IL-6 expression in RA patients. We found that levels of IL-6 were markedly higher in synovial fluids from patients with RA compared with those from OA patients (Fig. 1A). Analyses of tissue samples from the GEO database also revealed higher levels of IL-6 mRNA expression amongst RA patients than in OA patients (Fig. 1B), suggesting that IL-6 plays a more important role in RA than in OA.

### S1P facilitates IL-6 expression

**Figure 2:**
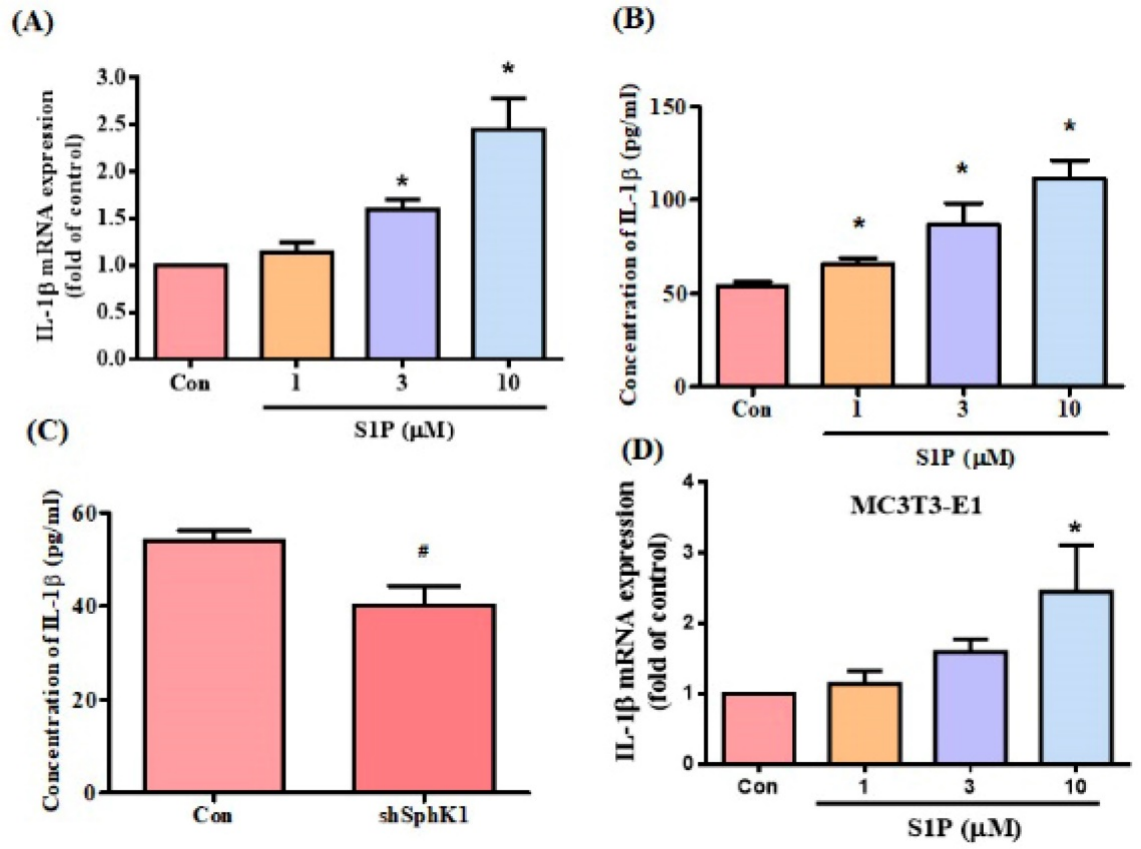
** S1P promotes IL-6 expression in osteoblasts.** MG-63 (A&B) and MC3T3-E1 (D) cells were incubated with S1P (1-10 μM) for 24 h; IL-6 expression was examined using the qPCR and ELISA assays. (C) MG-63 cells were transfected with SphK1 siRNA for 24 h; IL-6 expression was examined using the ELISA assay. Data represent the mean ± S.D. * *p* < 0.05 compared with the control group; # *p* < 0.05 compared with the control siRNA group.

S1P modulates osteoimmunology by targeting both osteoclastogenesis and osteogenesis 19. We sought to determine whether S1P also regulates IL-6 expression in osteoblasts (MG-63 cells). Stimulation of osteoblasts with S1P concentration-dependently promoted IL-6 mRNA expression and protein secretion (Fig. 2A&B). To confirm the role of S1P in IL-6 production, the cells were transfected with SphK1 siRNA, which diminished IL-6 expression (Fig. 2C). Similarly, the data from another osteoblast cell line (MC3T3-E1) showed that S1P increased IL-6 expression (Fig. 2D). S1P therefore appears to promote IL-6 production in osteoblasts.

### S1P promotes IL-6 expression through the PI3K and MEK/ERK pathways

**Figure 3:**
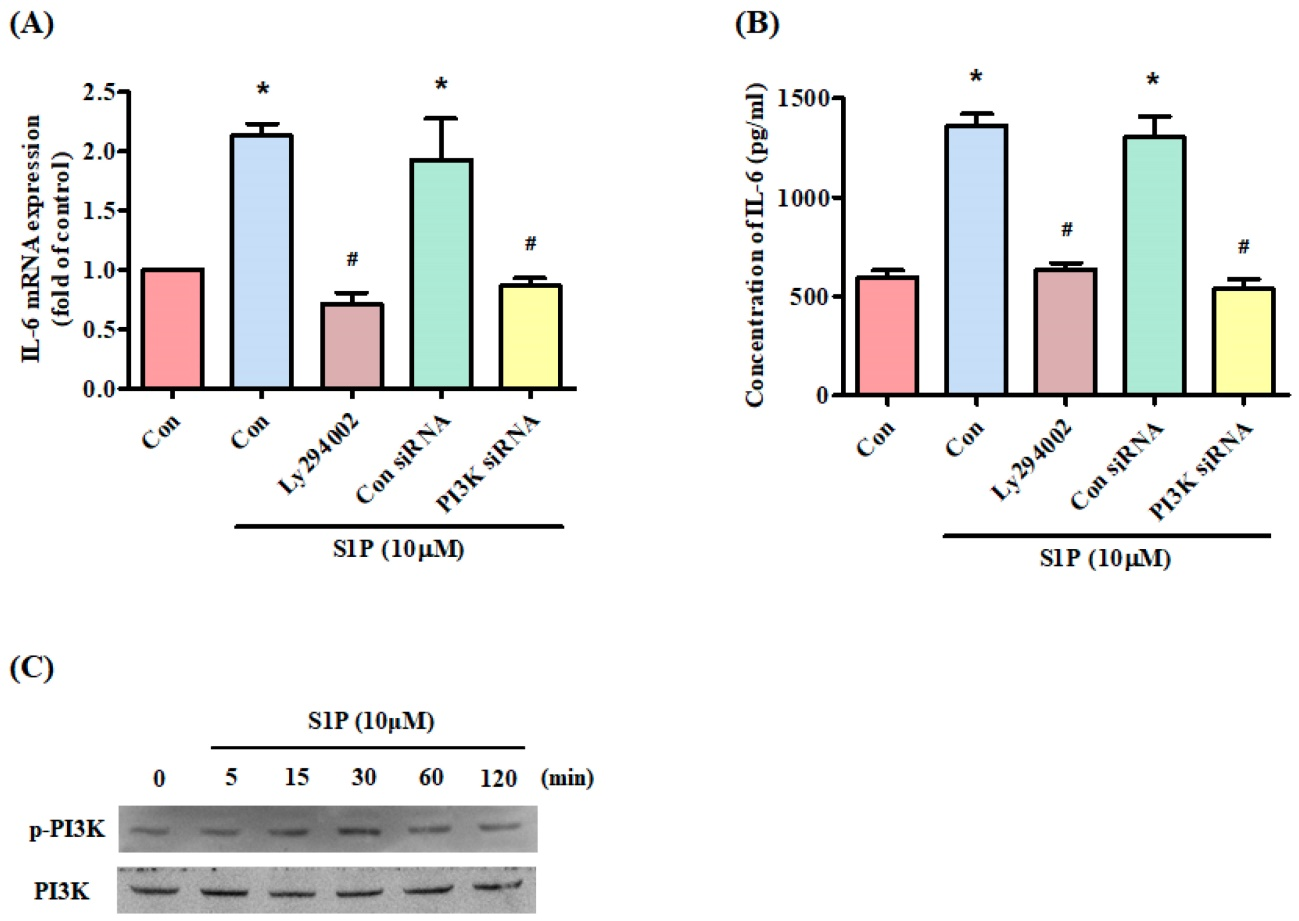
** The PI3K pathway mediates S1P-promoted IL-6 expression.** (A&B) MG-63 cells were pretreated for 30 min with LY294002 or transfected with PI3K siRNA then stimulated with S1P. IL-6 expression was examined using the qPCR and ELISA assays. (C) MG-63 cells were incubated with S1P; PI3K phosphorylation was examined using the Western blot assay. Data represent the mean ± S.D. * *p* < 0.05 compared with the control group; # *p* < 0.05 compared with the S1P-treated group.

The PI3K signaling pathway controls IL-6 production 31. Treatment of cells with a PI3K inhibitor (Ly294402) reduced S1P-promoted IL-6 (Fig. 3A&B). Transfection of the cells with PI3K siRNA reversed S1P-mediated effects (Fig. 3A&B), while incubation of osteoblasts with S1P facilitated PI3K phosphorylation (Fig. 3C), indicating that PI3K is involved in S1P-enhanced IL-6 production.

**Figure 4:**
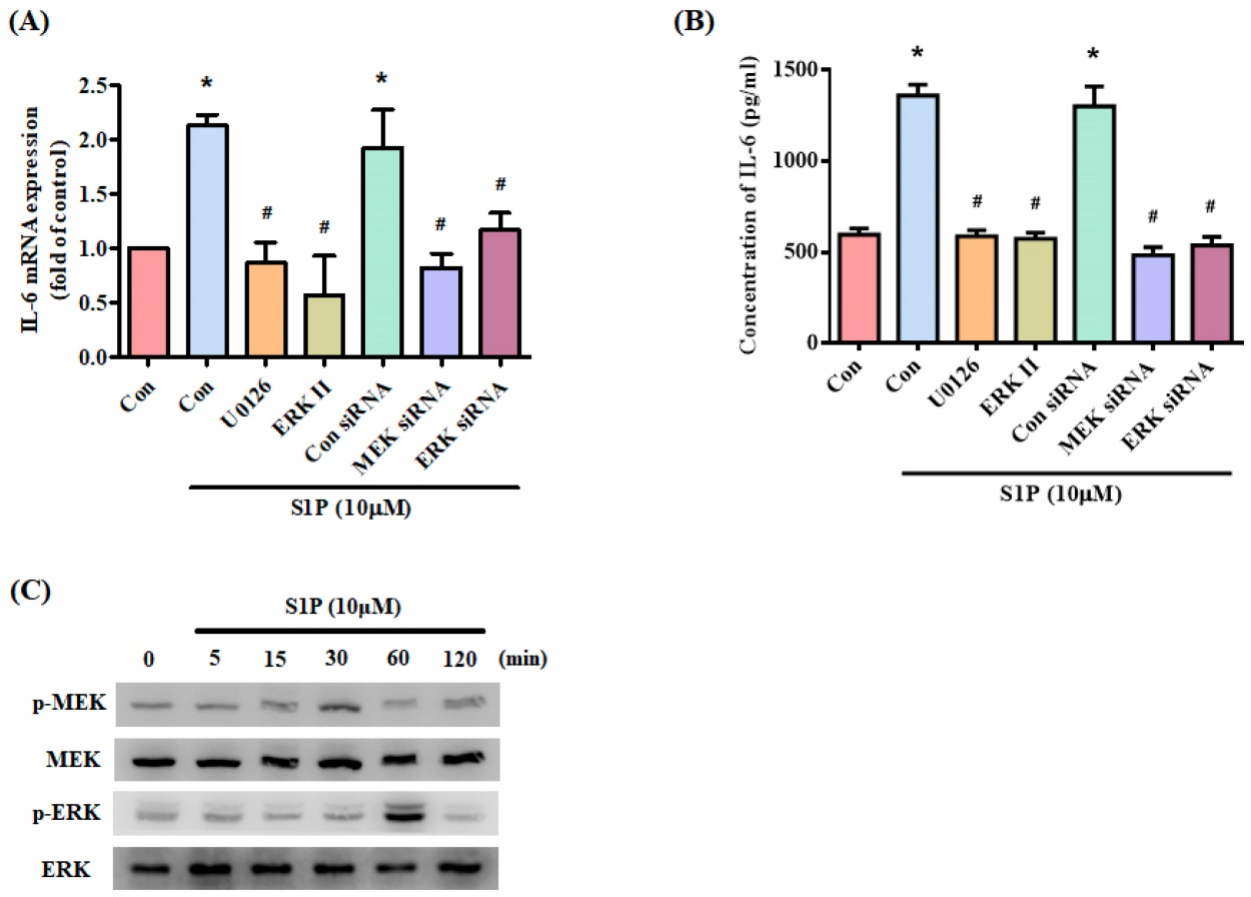
** The MEK/ERK pathway is involved in S1P-induced IL-6 expression.** (A&B) MG-63 cells were pretreated for 30 min with U0126 and ERK inhibitor or transfected with MEK and ERK siRNAs then stimulated with S1P. IL-6 expression was examined by qPCR and ELISA assays. (C) MG-63 cells were incubated with S1P; MEK and ERK phosphorylation was examined using the Western blot assay. Data represent the mean ± S.D. * *p* < 0.05 compared with the control group; # *p* < 0.05 compared with the S1P-treated group.

MEK/ERK is a common signaling pathway for enhancing IL-6 expression 32. Incubating osteoblasts with a MEK inhibitor (U0126) and ERK inhibitor or siRNAs against MEK and ERK effectively inhibited S1P-promoted IL-6 expression (Fig. 4A&B). Stimulation of osteoblasts by S1P increased MEK and ERK phosphorylation (Fig. 4C). These results suggest that S1P acts via PI3K and MEK/ERK signaling to enhance levels of IL-6 expression.

### NF-κB transcriptional activity controls S1P-induced IL-6 production

**Figure 5:**
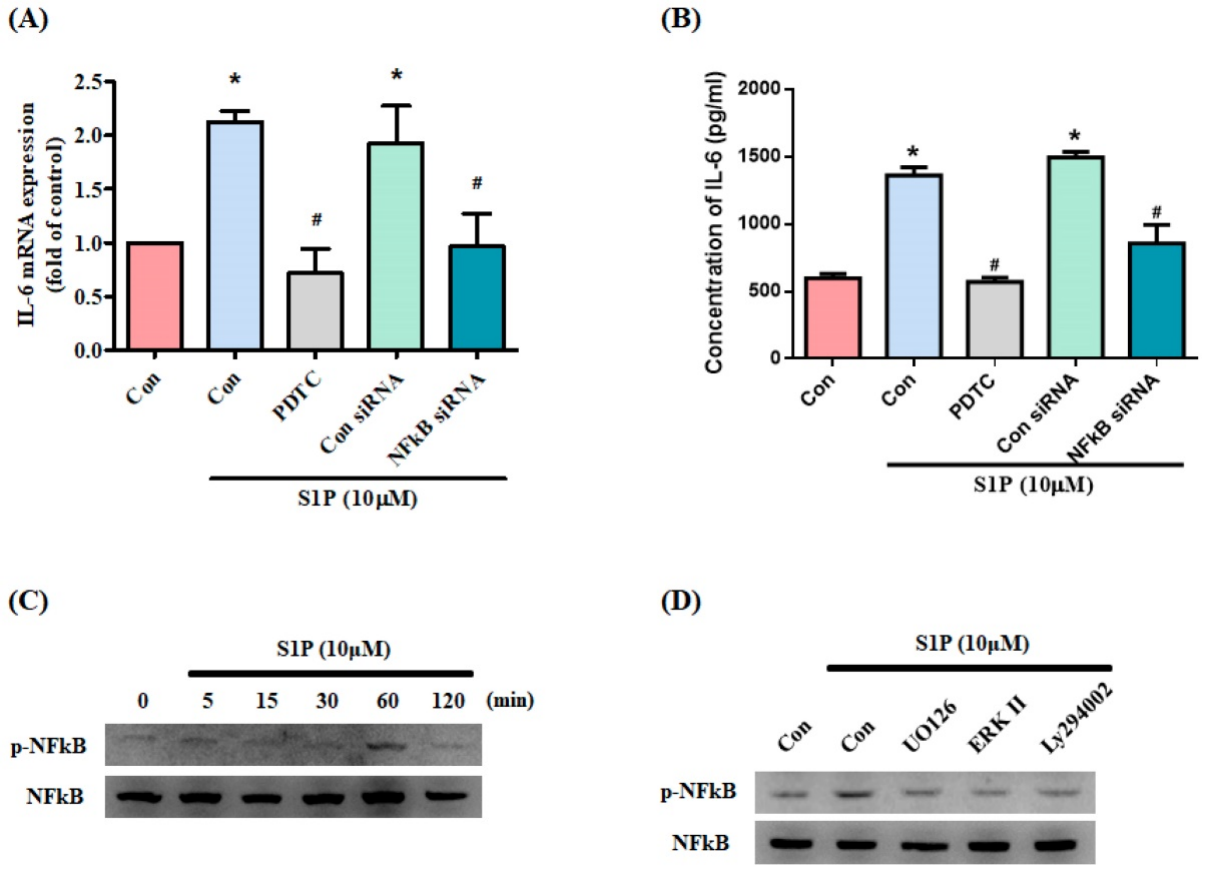
** S1P increases IL-6 production through NF-κB activation.** (A&B) MG-63 cells were pretreated for 30 min with PDTC or transfected with NF-κB siRNA then stimulated with S1P. IL-6 expression was examined by qPCR and ELISA assays. (C) MG-63 cells were incubated with S1P; NF-κB phosphorylation was examined using the Western blot assay. (D) MG-63 cells were pretreated with LY294002, U0126 or an ERK inhibitor for 30 min, then stimulated with S1P and NF-κB phosphorylation. Data represent the mean ± S.D. * *p* < 0.05 compared with the control group; # *p* < 0.05 compared with the S1P-treated group.

NF-κB appears to control *IL-6* gene regulation 33. We therefore examined whether NF-κB influences S1P-promoted IL-6 expression in osteoblasts. Treating cells with the NF-κB inhibitor PDTC or transfecting them with NF-κB siRNA inhibited S1P-induced IL-6 production (Fig. 5A&B). S1P significantly promoted NF-κB phosphorylation (Fig. 5C), which was reduced by pretreatment with PI3K, MEK and ERK inhibitors (Fig. 5D).

**Figure 6:**
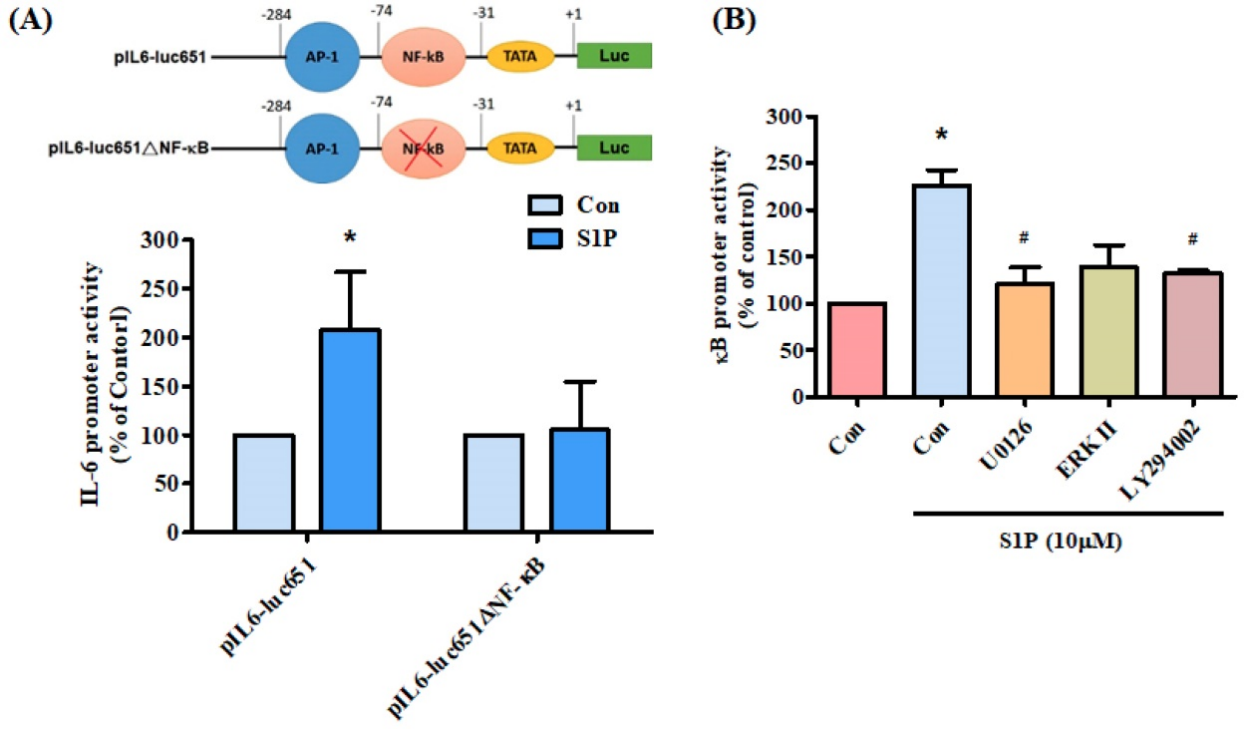
** PI3K, MEK and ERK pathways are involved in S1P-facilitated NF-κB activity.** (A) MG-63 cells were transfected with the indicated IL-6 luciferase plasmid and then stimulated with S1P. IL-6 luciferase activity was examined. (B) MG-63 cells were pretreated with LY294002, U0126 or an ERK inhibitor, then stimulated with S1P and NF-κB luciferase activity was examined. Data represent the mean ± S.D. * *p* < 0.05 compared with the control group; # *p* < 0.05 compared with the S1P-treated group.

We further examined the effect of the NF-κB transcriptional binding site in the induction of IL-6 expression, using the IL-6 promoter construct pIL6-luc651(-651/+1) and the NF-κB site mutation (pIL6-luc651ΔNF-κB). We found that S1P significantly increased pIL6-luc651 promoter activity, which was abolished by the NF-κB binding site mutation (Fig. 6A). To confirm that the PI3K and MEK1/ERK signaling pathway mediated S1P-enhanced activation of NF-κB, the NF-κB luciferase promoter plasmid was used. Treatment of cells with S1P augmented NF-κB luciferase activity, while pretreatment of the cells with PI3K, MEK and ERK inhibitors reduced S1P-induced NF-κB luciferase activity (Fig. 6B). Activation of PI3K MEK and ERK appears to be necessary for S1P-induced NF-κB activation in human osteoblasts.

## Discussion

RA is associated with chronic synovial inflammation and cartilage degradation in the joints. The chronic inflammatory progression in RA is regulated through multiple cytokine networks. The factors responsible for initiating the degradation and loss of the articular tissues are largely unknown. Upregulation of S1P has been found in synovial tissues of the collagen-induced arthritis model 20. However, little is known about the role of S1P in the expression of the critical inflammatory cytokine IL-6 and RA development. Here, we found that S1P increases IL-6 production in human osteoblasts through the PI3K, MEK/ERK and NF-κB signaling pathways.

IL-6 has numerous biological activities and previous research has revealed increased IL-6 concentrations in sera and synovial fluid from RA patients 34. In this study, we confirmed that IL-6 levels are higher in synovial fluids from RA patients than from OA patients. Our analysis of records from the GEO database also demonstrated higher IL-6 levels in RA tissue than in OA tissue. We also identified IL-6 as a target protein for the SIP signaling pathway, which regulates inflammatory responses in RA disease. These results highlight the critical nature of IL-6 as a molecular target in RA therapy.

Accumulating evidence focuses on the important role of inflammation in osteoblasts, which exhibit critical effects within the arthritic bone microenvironment and thus in RA pathogenesis 35, 36. The research suggests that the influence of inflammation in bone is specific to the site of inflammation and dependent on the cytokines present within the local bone microenvironment 35, 36. In this study, we found that S1P enhances IL-6 production in osteoblasts and that knockdown SphK1 diminishes IL-6 expression in osteoblasts. Our results provide evidence that inflammation in osteoblasts plays a critical role during the RA development.

The activation of the PI3K and MEK/ERK signaling pathway is essential for regulating multiple cellular functions 37. These pathways also regulate IL-6 expression 33, 38. In this study, we found that S1P facilitates PI3K, MEK and ERK phosphorylation, while PI3K, MEK and ERK inhibitors diminish S1P-enhanced IL-6 production. Furthermore, PI3K, MEK and ERK siRNAs confirmed the reversal of S1P-promoted IL-6 expression. It appears that the PI3K and MEK/ERK signaling pathways are involved in S1P-regulated IL-6 expression. Several binding sites exist for a number of transcription factors in the 5' region of the *IL-6* gene 39, 40. The results of this study show that a mutation of the NF-κB binding site inhibited S1P-enhanced IL-6 luciferase activity. In addition, an NF-κB inhibitor or siRNA reversed S1P-induced IL-6 expression. These results indicate that NF-κB activation is required for S1P-promoted IL-6 production. Our data also show that S1P facilitates NF-κB phosphorylation and luciferase activity. PI3K, MEK and ERK inhibitors all antagonized S1P-enhanced NF-κB phosphorylation and luciferase activity, suggesting that the PI3K, MEK and ERK pathways regulate S1P-mediated NF-κB activation.

**Figure 7:**
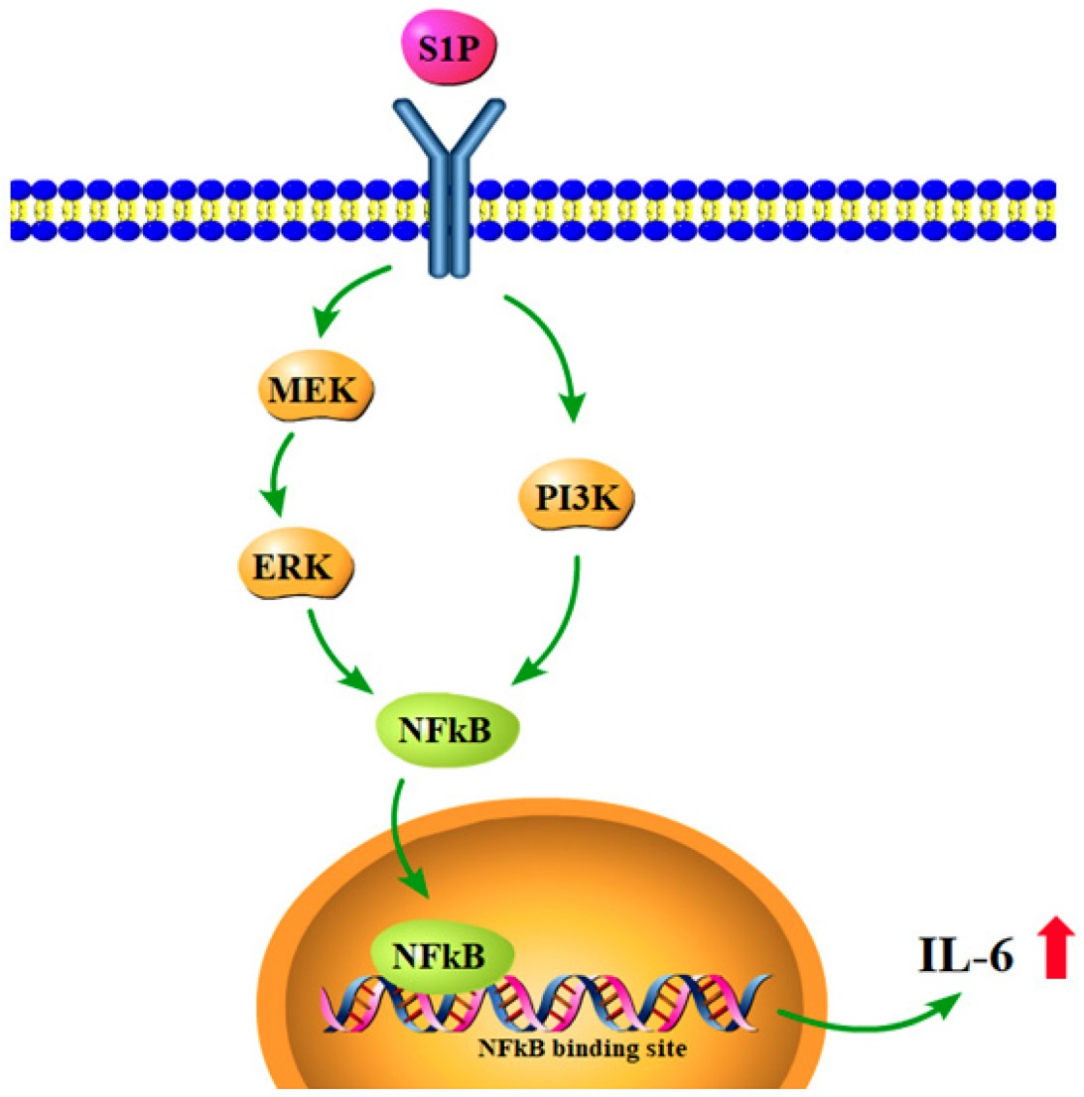
** Schematic diagram summarizes the mechanisms of S1P-induced IL-6 production in osteoblasts.** S1P promotes IL-6 expression in osteoblasts through the PI3K, MEK/ERK and NF-κB signaling pathways.

In conclusion, our study has identified that S1P promotes IL-6 production and inflammatory responses in osteoblasts via the PI3K, MEK/ERK and NF-κB signaling pathways (Fig.7). S1P appears to be a novel therapeutic target in RA.
